# Mobile Phone Apps for Intimate Partner and Sexual Violence Prevention and Response: Systematic Search on App Stores

**DOI:** 10.2196/28959

**Published:** 2022-02-08

**Authors:** Jessica Draughon Moret, Angela Todd, Lauren Rose, Erin Pollitt, Jocelyn Anderson

**Affiliations:** 1 Betty Irene Moore School of Nursing University of California, Davis Sacramento, CA United States; 2 College of Nursing Pennsylvania State University University Park, PA United States; 3 District of Columbia Forensic Nurse Examiners Washington, DC United States

**Keywords:** rape, intimate partner violence, gender-based violence, smartphone, mobile phone app

## Abstract

**Background:**

Since the 2008 advent of the smartphone, more than 180 billion copies of apps have been downloaded from Apple App Store, with more than 2.6 million apps available for Android and 2.2 million apps available for iOS. Many violence prevention and response apps have been developed as part of this app proliferation.

**Objective:**

This study aims to evaluate the prevalence and quality of freely available mobile phone apps targeting intimate partner violence (IPV) and sexual violence (SV) prevention and response.

**Methods:**

We conducted a systematic search of violence prevention and response mobile phone apps freely available in Apple App Store (iOS; March 2016) and Google Play Store (Android; July 2016). Search terms included violence prevention, sexual assault, domestic violence, intimate partner violence, sexual violence, forensic nursing, wife abuse, and rape. Apps were included for review if they were freely available, were available in English, and had a primary purpose of prevention of or response to SV or IPV regardless of app target end users.

**Results:**

Using the Mobile Application Rating Scale (MARS), we evaluated a total of 132 unique apps. The majority of included apps had a primary purpose of sharing information or resources. Included apps were of low-to-moderate quality, with the overall subjective quality mean for the reviewed apps being 2.65 (95% CI 2.58-2.72). Quality scores for each of the 5 MARS categories ranged from 2.80 (engagement) to 4.75 (functionality). An incidental but important finding of our review was the difficulty in searching for apps and the plethora of nonrelated apps that appear when searching for keywords such as “rape” and “domestic violence” that may be harmful to people seeking help.

**Conclusions:**

Although there are a variety of mobile apps available designed to provide information or other services related to SV and IPV, they range greatly in quality. They are also challenging to find, given the current infrastructure of app store searches, keyword prioritization, and highlighting based on user rating. It is important for providers to be aware of these resources and be knowledgeable about how to review and recommend mobile phone apps to patients, when appropriate.

## Introduction

Both sexual violence (SV) and intimate partner violence (IPV) continue to be major public health problems in the United States and worldwide. Every 68 seconds, a US resident is sexually assaulted [1]. In the 2015 National Intimate Partner and Sexual Violence Survey, 1 in 3 (33%) people reported experiencing lifetime physical violence or SV [2]. When psychological abuse is considered, the numbers are closer to 1 in 2 (50%) people. Rates are often higher in lesbian, gay, bisexual, transgender, and queer (LGBTQ) populations, with 50% of transgender people, 61% of bisexual women, 44% of lesbian women, 37% of bisexual men, and 26% of gay men reporting an experience of IPV in their lifetime [3,4].

Documented direct and indirect health outcomes linked to IPV and SV include physical, mental, and sexual health sequelae. In addition to physical injury, these types of violence are associated with chronic stress, chronic immune system activation, and inflammation [5]; accelerated cellular aging [6]; and cardiovascular disease risk [7]. Depression, acute stress disorder, and posttraumatic stress disorder are common comorbidities [8]. IPV and SV are also associated with substance use, alcohol use, and sexual risk taking, all of which are documented risk factors for HIV and other sexually transmitted infections. In addition, experience of SV and IPV is associated with diminished control over sexual and reproductive health decisions [9], unplanned pregnancies, preterm labor, low-birthweight babies, and maternal morbidity and mortality [10].

Although rates of SV and IPV have remained relatively stable over the past decade, the ways that people access and gather information have changed. Social media and other mobile apps are most people’s preferred source of information [11]. Smartphones are ubiquitous among adolescents and young adults, with 98% of Generation Z owning a smartphone [12]—overlapping with those at highest risk for sexual assault ages 12-34 years [13]. As researchers and clinicians working on SV and IPV, we recognized this shift toward internet- and smartphone-available information was imminent in our field. For example, in 2012, early media reports of the UAskDC app garnered attention, showing that these spaces were being utilized and that information in apps could be vetted in partnership with reputable health and advocacy service providers [14-16].

The UAskDC app effectively curated the many disparate resources from each higher education campus Title IX and student affairs office, and health, advocacy, and criminal justice services across the District of Columbia into 1 place. The app provides more accurate and trauma-informed information than a Google search for “rape” or “sexual assault” and “District of Columbia” would, and the platform allows for rapid updates. Resource sharing apps, such as UAskDC, are focused on secondary and tertiary prevention—connecting a survivor to resources for safety and health. These community-specific apps, while designed for potential survivors/patients, are also an invaluable resource for health care providers, friends, and family members who may be trying to direct a patient or loved one to appropriate resources. Because most health care providers are not experts in IPV or SV, receiving an average of only 1-5 hours of training in these topics during their prelicensure training [17], resources used by health systems (eg, handouts, apps, and websites) become heavily-relied-upon sources of information. Therefore, it is of utmost importance that the quality of these apps be known and maintained to achieve their goals.

Although IPV and SV prevention and response apps are widely available, the literature focused on these apps remains limited. Of the studies including IPV- or SV-related apps, most examined apps that were directed at college-aged women offering resources for use during or after SV to support safety and decision making [18-21], while 2 were directed toward education in recognition and prevention of child sexual abuse and trafficking [22,23]. Overall, these studies found potential for IPV- or SV-related apps to educate users about prevention, recognition, harm reduction, safety measures, and resources for victims of IPV or SV [18-23].

Perhaps the most documented IPV app in the literature is MyPlan [24]. MyPlan draws on elements of social cognitive and decision-making theories through self-monitoring, social support, and priority setting [25]. MyPlan further integrates safety-planning strategies and tools used by IPV advocates for decades [26,27]. This app allows survivors to evaluate their relationship and safety while designing a plan tailored to their individual needs and simultaneously receiving resources with embedded links. It allows survivors to return to their plan and review and update information over time to coincide with changes within their abusive relationship. In prospective clinical trials, MyPlan and its precursor, the computer-based decision aid Internet Resource for Information and Safety (IRIS), both found improvements in decisional conflict, use of relationship safety strategies, and ending unsafe relationships [28-30].

Given the proliferation of apps and our prior experience developing and testing a mobile app for IPV and SV response, we are aware of the multiple challenges with app dissemination and maintenance [20]. Therefore, this paper aims to determine the prevalence and quality of freely available mobile smartphone apps that include a primary goal of addressing prevention and response. A secondary aim was to determine priority recommendations for health care providers interested in integrating mobile apps within patient care.

## Methods

### Study Design

We conducted a search of Apple App Store (March 2016) and Google Play Store (July 2016) using the following search terms: violence prevention, sexual assault, domestic violence, intimate partner violence, sexual violence, forensic nursing, wife abuse, and rape. Complete lists of results were downloaded to Microsoft Excel for review. SV and IPV apps were included in this analysis as they both commonly co-occur (approximately 18% of women and 8% of men report lifetime intimate partner sexual violence [31]) and are commonly addressed by services that are colocated or multipurpose (eg, a community’s IPV shelter also provides rape crisis accompaniment services to health care facilities).

Titles were reviewed by a member of the study team to determine whether inclusion criteria were met. When the title was unclear, they continued to the next step, which was review of the app’s general information available in the publisher’s app store without downloading the app. For apps that clearly met the inclusion criteria or in which it was unclear from the information available in the app store Information section, we continued to the final step: full review via download of the app to a mobile phone or tablet.

Inclusion criteria for our analysis were (1) available in English, (2) free version available, and (3) directed toward 1 of the following audiences: the general public at risk for SV or IPV, people who have experienced IPV or SV, or health or advocacy providers who work with people who experience violence.

### App Review

Apps were reviewed for quality using the Mobile Application Rating Scale (MARS) [32]. Since its initial publication in 2015, MARS has been used to evaluate the quality of smartphone apps on a wide range of health-related topics. These include health promotion topics, such as fitness [33], nutrition and weight management [34-36], mental health [37], and mindfulness [38], as well as self-management of medical conditions, such as diabetes [39], sleep disorders [40], pain management [41], heart failure [42], and asthma [43].

For this analysis, modifications to MARS were made to ensure fit for the SV and IPV content area. These included using “sexual or intimate partner violence” to fill in the content areas for the target health behavior in the Perceived Impact of Health Behavior Change section, including instructions for categorizing violence advocacy and service agencies when addressing the item on credibility, and adding features that we knew to be potentially common or relevant to the goals of violence-specific apps (eg, Global Positioning System [GPS], linking to service providers, emergency exit features). Our full data collection instrument is available in Multimedia Appendix 1.

All data were entered into an internet-based survey form, which also collected date and time information as well as which research team member was entering the data. At the search onset, the team selected 4 apps to all independently review and discuss during a team meeting to create shared definitions and consistency within the team. Subsequently, each app was reviewed by 1 team member with consultation to the team, as needed. Apple platform apps were reviewed between April 2016 and September 2016, and Google platform apps were reviewed between October 2017 and February 2018.

### Data Analysis

Descriptive analyses were completed to summarize the apps reviewed. Mean scores were calculated for each of the MARS categories (engagement, functionality, aesthetics, information, and subjective quality). Individual item frequencies and proportions were also calculated for nonscale items. Data were analyzed using SPSS Statistics version 25 (IBM Corp.) [44]. App classification data were recoded by consensus of 2 of the team members (authors JCA and LR) to better summarize results and due to the large number of “Other” responses in some categories (initial *n*=38, 28.8% for focus area; *n*=14, 10.6% for theoretical background/strategies) based on the initial use of MARS with minor modifications.

## Results

### Search Results

**Figure 1:**
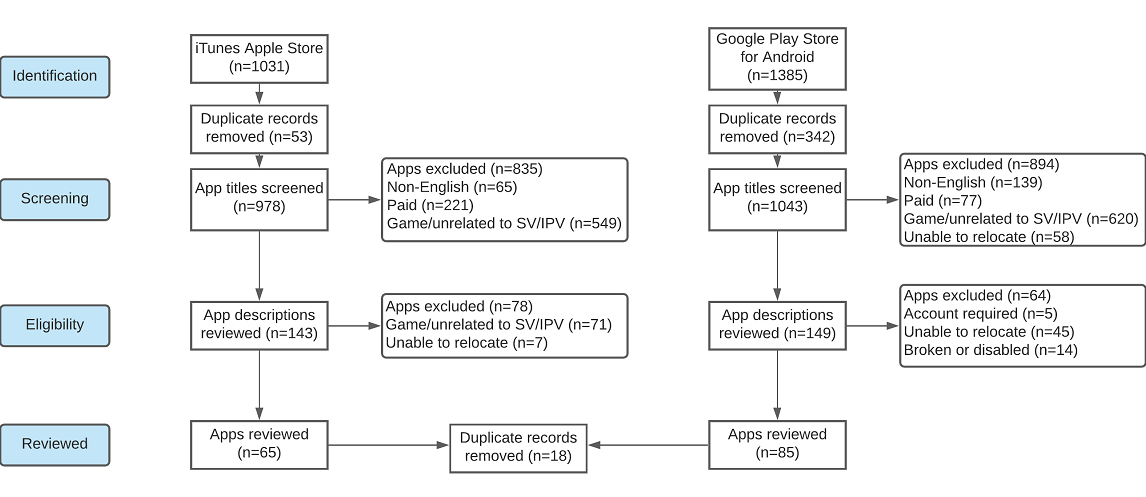
Flow diagram of the app search and inclusion process. IPV: intimate partner violence; SV: sexual violence.

In our initial searches, 978 unique apps were identified from Apple App Store and 1043 from Google Play Store. Of the app titles screened, 835 (85.4%) Apple apps and 894 (85.7%) Google apps were excluded (see Figure 1 for the search flow diagram), resulting in 143 (14.6%) Apple apps and 149 (14.3%) Google apps for store description abstract information review. Following this step, 65 (45.5%) Apple and 85 (57%) Google apps met the inclusion criteria and remained for full analysis. Of these 150, 18 (12%) apps appeared in both app stores, and duplicates were removed from the list, resulting in 132 (86%) apps in the final analysis.

Although it was not an aim of this project, an additional important finding came to light during the app store search process. Unlike typical search engines (eg, Google) or research databases (eg, PubMed), Boolean operators (“and,” “or”) do not work when searching in app stores. Searching in app stores is heavily reliant on developer tagging of titles and keywords, ratings and reviews from other users, and advertising/marketing monies spent to promote apps [45,46]. Because of how these search functions work, searching for the word “rape” in Apple App Store brings up hundreds of results for voice changer and rap music apps (presumably because of the similar spelling of “rape” and “rap”). Searching for sexual assault, synonymous but more formal terminology, in the same Apple App Store brings up only a fraction of the results and far more that are designed for SV providers, advocates, prevention, and response. These differences in search strategies and logic have important implications for providers who may be recommending use of apps to individuals they work with.

### Descriptives

Table 1 summarizes the apps’ targeted age groups, features, focus areas, behavior change strategies, and organizational affiliations. Over three-quarters (101/132, 76.4%) of the apps focused on resource or information sharing, with at least 10% of apps focusing on each of the following: IPV (37/132, 28%), crisis intervention or mental health (29/132, 22.2%), sexual assault (18/132, 13.6%), relationship conflict/health (17/132, 12.9%), and peer support (15/132, 11.4%). Apps primarily targeted adults (87/132, 65.9%), young adults (36/132, 27.3%), or no specific age group (31/132, 23.5%). In addition, 1 in 3 (45/132, 34.1%) apps was affiliated with a nonprofit or nongovernment agency and 1 in 4 (36/132, 27.3%) with a government agency. Nearly 1 in 5 (26/132, 19.7%) apps had developers or content that did not allow their affiliation to be determined. The most common features or functions observed in the reviewed apps included requiring internet service to operate (21/132, 15.9%), location or GPS services (20/132, 15.2%), and emergency exit/panic features (17/132, 12.9%); however, these were still only present in 13-20 (10%-15%) apps. Additional functions, such as logins, passwords, and reminders, were each present in a minority of apps. Apps overwhelmingly used information and education as a behavior change (104/132, 78.8%), with safety monitoring/tracking (15/132, 11.4%) and goal setting/safety planning (14/132, 10.6%), each following with approximately 1-10 (0.7%-7.6%) apps using these strategies.

**Table 1:** App overview information (N=132).

Category		Apps, n (%)
**App focus areas^a^**		
	Resource or information sharing	100 (76.4)
	IPV^b^	37 (28.0)
	Crisis intervention/mental health	29 (22.2)
	Sexual assault	18 (13.6)
	Relationship conflict/health	17 (12.9)
	Peer support	15 (11.4)
	General violence risk	9 (6.8)
	Behavior change	6 (4.5)
	Goal setting	6 (4.5)
	Physical health	3 (2.3)
	Safety planning	24 (18.2)
	Entertainment	1 (0.8)
	Education	4 (3.2)
	Legal	2 (1.6)
	Other	2 (1.6)
**Target age groups^a^**		
	General	31 (23.5)
	Adults	87 (65.9)
	Young adults	36 (27.3)
	Teens	13 (9.8)
	Children (<12 years)	2 (1.5)
**Affiliations**		
	Nongovernment organization/nonprofit	45 (34.1)
	Government organization	36 (27.3)
	Unknown	26 (19.7)
	University/educational organization	13 (9.8)
	Commercial organization	6 (4.5)
	Health care organization	2 (1.2)
**App features or functions^a^**		
	Web required	21 (15.9)
	Location services	20 (15.2)
	Panic/exit	17 (12.9)
	Social media sharing	9 (6.8)
	Login	8 (6.1)
	Password	7 (5.3)
	Reminders	6 (4.5)
	Integration with phone (eg, calendar or reminders)	2 (1.5)
	App community	1 (0.8)
**App theoretical background/intervention strategies^a^**		
	Information/education	104 (78.8)
	Safety monitoring/tracking	15 (11.4)
	Goal setting/safety planning	14 (10.6)
	Other	12 (9.1)
	Location tracking	10 (7.6)
	Decision making	8 (6.1)
	Feedback	3 (2.3)
	Assessment	2 (1.5)

^a^Categories are not mutually exclusive.

^b^IPV: intimate partner violence.

### App Quality

MARS classifies app quality into 5 categories: engagement, functionality, aesthetics, information, and overall subjective quality. The scale also includes a sixth domain of questions related to the perceived potential impact of an app on behavior change. App mean quality scores in this study ranged from 2.80 (engagement) to 4.75 (functionality), and the app perceived potential impact mean score was 3.02 (95% CI 2.84-3.20). The overall subjective quality mean for the reviewed apps was 2.65 (95% CI 2.58-2.72). The individual item means ranged from 1.08 (evidence base) to 4.15 (quantity of information), both items being within the information domain. Tables 2 and 3 summarize category and individual item scores for the reviewed apps.

**Table 2:** Mobile Application Rating Scale (MARS) app quality subscale ratings.

MARS section			Mean (SD)	95% CI
**Engagement**				
	Overall	2.08 (0.676)		1.96-2.20
	Entertainment	1.94 (0.935)		1.78-2.11
	Interest	2.14 (1.077)		1.95-2.33
	Customization	1.56 (0.981)		1.39-1.74
	Interactivity	1.54 (0.896)		1.38-1.70
	Target group	3.26 (0.734)		3.13-3.39
**Functionality**				
	Overall	3.73 (0.957)		3.57-3.90
	Performance	3.45 (1.163)		3.25-3.66
	Ease of use	3.95 (0.847)		3.80-4.10
	Navigation	3.95 (0.932)		3.79-4.12
	Gestural design	3.98 (0.704)		3.85-4.10
**Aesthetics**				
	Overall	3.38 (0.757)		3.25-3.52
	Layout	3.83 (0.853)		3.68-3.98
	Graphics	3.32 (0.829)		3.17-3.47
	Visual appeal	3.08 (0.771)		2.94-3.22
**Information**				
	Overall	2.71 (0.731)		2.58-2.84
	Accuracy of description	3.74 (1.023)		3.56-3.92
	Goals	2.15 (1.628)		1.86-2.44
	Quality of information	4.02 (1.402)		3.76-4.27
	Quantity of information	4.15 (1.186)		3.92-4.38
	Visual information	1.7 (1.453)		1.44-1.96
	Credibility	2.94 (1.148)		2.74-3.15
	Evidence base	1.08 (0.302)		1.03-1.13

**Table 3:** Mobile Application Rating Scale (MARS) app subjective quality ratings and perceived impact scores.

Subjective quality and perceived impact items		Mean (SD)	95% CI
**Subjective quality**			
	Overall score	2.65 (0.398)	2.58-2.72
	Would you recommend this app to people who might benefit from it?	3.37 (1.147)	3.16-3.57
	How many times do you think you would use this app in the next 12 months if it was relevant to you?	1.62 (0.778)	1.48-1.76
	What is your overall star rating of the app?	3.06 (0.905)	2.90-3.22
**Perceived impact**			
	Overall score	3.02 (1.005)	2.84-3.20
	Awareness: This app is likely to increase awareness of the importance of sexual assault and IPV^a^.	2.97 (1.101)	2.77-3.16
	Knowledge: This app is likely to increase knowledge/understanding of sexual assault and IPV.	2.96 (1.165)	2.75-3.17
	Attitudes: This app is likely to change attitudes toward improving sexual assault and IPV.	3.34 (1.043)	3.15-3.52
	Intention to change: This app is likely to increase intentions/motivation to address sexual assault and IPV.	3.11 (1.061)	2.92-3.30
	Help seeking: Use of this app is likely to encourage further help seeking for sexual assault and IPV (if it is required).	2.73 (1.188)	2.52-2.94
	Behavior change: Use of this app is likely to increase/decrease sexual assault and IPV (of their sequelae).	3.10 (1.032)	2.91-3.28

^a^IPV: intimate partner violence.

## Discussion

### Principal Findings

Despite reviewing over 100 freely available, English language mobile apps targeted at SV and IPV prevention and response, the overall quality was average. There were few apps that we ourselves as experienced forensic examiners would use as clinicians or recommend to our patients after a physical or sexual assault. We recognize the limitations of a dated search in a rapidly evolving mobile app space. However, our primary findings related to (1) identifying relevant apps and (2) high-quality evidence-based apps remain salient.

The focus of the apps was largely on education and information sharing. Although individuals are spending more time on mobile phones, if the focus is largely on education and information sharing, we fear that these apps will not be successful in meeting their desired goal. As with any health promotion and prevention content, mobile app content must be regularly reviewed, and updated for accuracy—and the mobile app platform adds additional technology hurdles to overcome regarding maintaining the infrastructure and content in ways that are accessible and engaging for users. Although not part of any of the MARS subscales, the tool does include collecting data on the number of times an app was rated and the current app rating. Of the 132 included apps, 104 (78.8%) had at least 1 rating listed (median user rating across rated apps was 4.20; however, the median number of user ratings across rated apps was 2, with a range of 1 to >21,000). These variations in how apps are marketed, downloaded, and shared among networks highlight 1 key area of their usefulness and 1 challenge in their dissemination in violence prevention and response work [20,47].

Marketing and sharing are key variables in how app-sharing platforms disseminate content to users and are not necessarily a skill set that violence advocates and health care providers have been trained in or possess. Notably, we believe the most concerning finding of this search was incidental. We were disturbed during the search process at the juxtaposition of violence prevention and response apps with zombie-killing games (any search including the term “violence”), dating sims (“intimate”), and the aforementioned voice changer app (when searching “rape”). The potential for retraumatization of our patients if they search these app stores looking for appropriate resources is high.

### Recommendations for Health Care Providers

Clinicians caring for patients after sexual or physical violence interested in sharing a mobile app–based resource with their patient population should treat any app similar to any other resource. The resource should be vetted by the health care team before adoption. As things in the mobile app industry change at an ever-increasing pace, any recommended app should routinely be reviewed to ensure it is still up to date and has not gone defunct. Several of the apps we identified in the initial search were not available by the time we returned to review them (see Figure 1).

Clinicians interested in providing mobile app resources to their patients experiencing IPV or SV should consider preidentifying a few select mobile app resources and sharing them directly with interested patients via a QR code or direct link to prevent patients from searching the app stores on their own. This would reduce the potential for retraumatization related to inappropriate or unexpected results of a search of the major app stores. Alternatively, if the patient allows or prefers, providers could search for and download the app directly onto the patient’s phone or device on their behalf.

Clinicians interested in developing and launching a mobile health app for their target population should consider using or adopting an existing tool versus creating a new one. The costs of developing and maintaining an app must be weighed against the other services that could be rendered with those funds. There are costs associated with creation and design, as well as costs to publish an app on each individual platform and maintenance costs to ensure the app remains relevant [48]. One piece of data that may have supported this in our analysis was the large number of apps that were initially found in our title searches but were not able to be relocated by the time we undertook our full analysis. There are multiple reasons that apps are removed from app stores. Primary reasons are related to apps not being compatible with current hardware or software requirements. As technology moves extraordinarily rapidly, maintaining apps requires diligent attention to these requirements to stay current. Our results were also consistent with a 2016 examination of the turnover of mental health apps, which found that in approximately a 6-month period, there was a 50% turnover (eg, apps were found on the initial search and not found on subsequent searches) in search results on the Android platform, whereas in iOS, approximately 90% of apps remained in the app store throughout the entire 9-month study period [49].

### Recommendations for Research

Few of the apps included had any scholarship or evidence associated with their effectiveness. It is difficult to recommend an app for use in a clinical setting when there is no evidence related to whether it achieves its stated goals. Many of the apps were targeted at information sharing; something as simple as a test-retest knowledge assessment would provide at least basic data regarding whether the app is effective in increasing knowledge. There is also a precedent for evaluating mobile apps in their target population as well as with relevant service providers [20,21,29,50-54].

As we discovered during our analysis, MARS may not be the best tool for evaluating violence prevention and response apps. Although we adapted the tool for our use, we would recommend further adjustments in the future. For example, several of the features noted anecdotally may be worth formally evaluating (eg, how and when GPS is integrated into the app, the presence of an “emergency exit” button). It would also be prudent to assess and understand the limits of data confidentiality, as GPS can be used by apps to assist people in help seeking but also by IPV perpetrators to track their victims. We also did not further adapt MARS for items such as whether the apps used a trauma-informed approach [55]. Factors that may make an app useful to a provider or patient who has experienced violence, such as whether it is designed with trauma-informed principles in mind (eg, is the information not only correct but also written using language that is nonjudgmental and easily understandable during a traumatic situation), are not currently captured in MARS and would be beneficial to include in future work on violence and trauma-related mobile apps.

There is also the continued difficulty of many apps placing the onus of violence prevention on the potential victim. Many apps are dependent on a potential victim taking a precautionary behavior: downloading the app, setting up a network, and holding a button on an app down until they are “safe.” These types of interventions perpetuate victim blaming, both blaming by others and self-blaming. Blaming is a form of retraumatization, which is in direct conflict with providing trauma-informed care [55].

### Limitations

Mobile apps and the mobile space are changing rapidly. The amount of time people spend on mobile apps increased by 35% in 2019 [12]. Unfortunately, health care research has historically moved at a much slower pace, and conducting a systematic search and analysis took an incredible investment of time. Based on our own data, by the time these data exist in the world, many of the included apps will likely no longer be accessible to the public, demonstrating the incredibly fast nature of how mobile apps come and go compared to how research is conducted. Conducting a search of a constantly changing medium required adjustment to traditional methods. We were unable to evenly divide up the apps for review due to device and platform availability at our respective institutions.

Additional limitations of this review included both the limitations inherent to the MARS tool and specifically its usefulness as a tool for evaluating violence apps. Although MARS standardizes language (eg, “This app is likely to increase awareness of the importance of address [insert target health behavior]), this still requires a reviewer to make numerous subjective decisions and assumptions. MARS also does not contain violence-specific content. This presented challenges in completely evaluating the aspects of apps that violence victims, survivors, or providers may find most important.

A final significant limitation is the often overlapping yet distinct needs of the people who interact with violence apps. Providers, friends, family, and survivors may all benefit from rapid collated access to local service information, but survivors may additionally want, need, or benefit from specific guided planning and resources. Providers or friends and family using an app to assist a patient or loved one may instead find the most benefit from tailored educational information and trauma-informed response information [19,56]. Although we broadly included all apps for these audiences in our search and analysis, we did not collect data to determine which apps appeared to specify which target audiences.

### Conclusion

In assessing freely available smartphone apps related to SV and IPV prevention or response, we note first the incredible amount of information that one needs to sift through before even getting to relevant apps. Over 2000 titles were assessed, including first-person shooter games and voice changer apps. Once narrowed to the 132 relevant and included apps, we must highlight that despite the number of apps in this space, the lack of quality and evidence base leaves much work to be done. As with any other item in our toolbox as health care providers and advocates, apps are 1 tool and will likely be most useful when implemented in the correct settings and with the appropriate knowledge, training, and skill sets.

## Appendix

Multimedia Appendix 1 Adapted Mobile App Rating Scale (MARS).

## Abbreviations

GPS: Global Positioning System
IPV: intimate partner violence
IRIS: Internet Resource for Information and Safety
LGBTQ: lesbian, gay, bisexual, transgender, and queer
MARS: Mobile Application Rating Scale
SV: sexual violence
