# Supplementary material for: Mobile Phone Apps for Intimate Partner and Sexual Violence Prevention and Response: Systematic Search on App Stores
Source: JMIR Form Res. 2022 Feb 8;6(2):e28959. doi: 10.2196/28959 (PMC8864520; doi:10.2196/28959)
Supplement: Multimedia Appendix 1 [file formative_v6i2e28959_app1.pdf]

Mobile App Rating Scale (Adapted)

Adapted from: Stoyanov S, Hides L, Kavanagh D, Zelenko O, Tjondronegoro D, Mani M
Mobile App Rating Scale: A New Tool for Assessing the Quality of Health Mobile Apps
JMIR Mhealth Uhealth 2015;3(1):e27
URL: https://mhealth.jmir.org/2015/1/e27
DOI: 10.2196/mhealth.3422

App Classification

The Classification section is used to collect descriptive and technical information about the app. Please review the app description in iTunes / Google Play to access this information.

App Name:

Platform

- ○ iPhone (1)
- ○ iPad (2)
- ○ Android (3)

Brief Description from the App Store (1st couple of lines)

Focus: what the app targets (select all that apply)

- ▢ Behavior Change (1)
- ▢ Goal Setting (2)
- ▢ Entertainment (3)
- ▢ Relationships (4)
- ▢ Physical Health (5)
- ▢ Peer Support (6)
- ▢ Safety Planning (7)
- ▢ Sexual Assault (8)
- ▢ Intimate Partner Violence (9)
- ▢ Risk of Violence (general) (10)
- ▢ Crisis Intervention (11)
- ▢ Resources (12)
- ▢ Other (13)

Q57 If "Other" please describe:

____

Theoretical background/strategies (select all that apply)

- ☐ Assessment (1)
- ☐ Feedback (2)
- ☐ Information/Education (3)
- ☐ Location Tracking (4)
- ☐ Safety Monitoring/Tracking (5)
- ☐ Goal Setting/Safety Planning (6)
- ☐ Advice/Tips/Strategies/Skill training (7)
- ☐ Decision Making (8)
- ☐ Other (9)

Q58 If "Other" please describe:

____

Affiliations

- ○ Unknown (1)
- ○ Commercial (2)
- ○ Government (3)
- ○ NGO (4)
- ○ University (5)
- ○ Hospital/Healthcare Organization/Health Affiliate (6)

Age group (select all that apply)

- ☐ Children (under 12) (1)
- ☐ Adolescents (13-17) (2)
- ☐ Young Adults (18-25) (3)
- ☐ Adults (4)
- ☐ General (5)

Technical aspects of app (all that apply)

- ☐ Allows sharing (Facebook, Twitter, etc.) (1)
- ☐ Has an app community (2)
- ☐ Allows password-protection (3)
- ☐ Requires login (4)
- ☐ Sends reminders (5)
- ☐ Needs web access to function (6)
- ☐ Panic/exit feature (7)
- ☐ Location Services (8)
- ☐ Integrates with other features/apps on device (9)

End of Block: App Classification

The Rating scale assesses app quality on four dimensions. Please use the descriptors provided for each response category.

Section A: Engagement – fun, interesting, customizable, interactive (e.g. sends alerts, messages, reminders, feedback, enables sharing), well-targeted to audience

---

Q1
Engagement: Is the app fun/entertaining to use? Does it use any strategies to increase engagement through entertainment (e.g. through gamification)?

- ○ Dull, not fun or engaging at all (1)
- ○ Mostly Boring (2)
- ○ OK, fun enough to engage user for a brief time (< 5 minutes) (3)
- ○ Moderately fun and entertaining, would engage user for some time (5-10 minutes total) (4)
- ○ Highly entertaining and fun, would stimulate repeated use (5)

Q2
Interest: Is the app interesting to use? Does it use any strategies to increase engagement by presenting its content in an interesting way?

- ○ Not interesting at all (1)
- ○ Mostly uninteresting (2)
- ○ OK, neither interesting nor uninteresting; would engage user for a brief time (< 5 minutes) (3)
- ○ Moderately interesting, would engage user for some time (5-10 minutes total) (4)
- ○ Very Interesting, would engage user in repeat use (5)

Q3
Customisation: Does it provide/retain all necessary settings/preferences for apps features (e.g. sound, content, notifications, etc.)?

- ◯ Does not allow any customisation or requires setting to be input every time (1)
- ◯ Allows insufficient customisation limiting functions (2)
- ◯ Allows basic customisation limiting functions (3)
- ◯ Allows numerous options for customisation (4)
- ◯ Allows complete tailoring to the individual's characteristic/preference, retains all settings (5)

Q4
Interactivity: Does it allow user input, provide feedback, contain prompts (reminders, sharing options, notifications, etc.)? Note: these functions need to be customisable and not overwhelming in order to be perfect.

- ◯ No interactive features and/or no response to user interaction (1)
- ◯ Insufficient interactivity, or feedback, or user input options, limiting functions (2)
- ◯ Basic interactive features to function adequately (3)
- ◯ Offers a variety of interactive features/feedback/user input options (4)
- ◯ Very high level of responsiveness through interactive features/feedback/user input options (5)

Q5
Target group: Is the app content (visual information, language, design) appropriate for your target audience?

- ◯ Completely inappropriate/unclear/confusing (1)
- ◯ Mostly inappropriate/unclear/confusing (2)
- ◯ Acceptable but not targeted. May be inappropriate/unclear/confusing (3)
- ◯ Well-targeted, with negligible issues (4)
- ◯ Perfectly targeted, no issues found (5)

Section B: Functionality – app functioning, easy to learn, navigation, flow logic, and gestural design of app

---

Q6
Performance: How accurately/fast do the app features (functions) and components (buttons/menus) work?

- ◯ App is broken, no/insufficient/inaccurate response (e.g. crashes/bugs/broken features, etc.) (1)
- ◯ Some functions work, but lagging or contains major technical problems (2)
- ◯ App works overall. Some technical problems need fixing/Slow at times (3)
- ◯ Mostly functional with minor/negligible problems (4)
- ◯ Perfect/timely response; no technical bugs found/contains a 'loading time left' indicator (5)

Q7
Ease of use: How easy is it to learn how to use the app; how clear are the menu labels/icons and instructions?

- ◯ No/limited instructions; menu labels/icons are confusing; complicated (1)
- ◯ Useable after a lot of time/effort (2)
- ◯ Useable after some time/effort (3)
- ◯ Easy to learn how to use the app (or has clear instructions) (4)
- ◯ Able to use app immediately; intuitive; simple (5)

Q8
Navigation: Is moving between screens logical/accurate/appropriate/ uninterrupted; are all necessary screen links present?

- ◯ Different sections within the app seem logically disconnected and random/confusing/navigation is difficult (1)
- ◯ Usable after a lot of time/effort (2)
- ◯ Usable after some time/effort (3)
- ◯ Easy to use or missing a negligible link (4)
- ◯ Perfectly logical, easy, clear and intuitive screen flow throughout, or offers shortcuts (5)

Q9
Gestural design: Are interactions (taps/swipes/pinches/scrolls) consistent and intuitive across all components/screens?

- ○ Completely inconsistent/confusing (1)
- ○ Often inconsistent/confusing (2)
- ○ OK with some inconsistencies/confusing elements (3)
- ○ Mostly consistent/intuitive with negligible problems (4)
- ○ Perfectly consistent and intuitive (5)

Section C: Aesthetics – graphic design, overall visual appeal, colour scheme, and stylistic consistency

Q10
Layout: Is arrangement and size of buttons/icons/menus/content on the screen appropriate or zoomable if needed?

- ○ Very bad design, cluttered, some options impossible to select/locate/see/read device display not optimized (1)
- ○ Bad design, random, unclear, some options difficult to select/locate/see/read (2)
- ○ Satisfactory, few problems with selecting/locating/seeing/reading items or with minor screen-size problems (3)
- ○ Mostly clear, able to select/locate/see/read items (4)
- ○ Professional, simple, clear, orderly, logically organised, device display optimised. Every design component has a purpose (5)

Q11 Graphics: How high is the quality/resolution of graphics used for buttons/icons/menus/content?

- ○ Graphics appear amateur, very poor visual design - disproportionate, completely stylistically inconsistent (1)
- ○ Low quality/low resolution graphics; low quality visual design – disproportionate, stylistically inconsistent (2)
- ○ Moderate quality graphics and visual design (generally consistent in style) (3)
- ○ High quality/resolution graphics and visual design – mostly proportionate, stylistically consistent (4)
- ○ Very high quality/resolution graphics and visual design - proportionate, stylistically consistent throughout (5)

Q12 Visual appeal: How good does the app look?

- ◯ No visual appeal, unpleasant to look at, poorly designed, clashing/mismatched colours (1)
- ◯ Little visual appeal – poorly designed, bad use of colour, visually boring (2)
- ◯ Some visual appeal – average, neither pleasant, nor unpleasant (3)
- ◯ High level of visual appeal – seamless graphics – consistent and professionally designed (4)
- ◯ As above + very attractive, memorable, stands out; use of colour enhances app features/menus (5)

Section D: Information – Contains high quality information (e.g. text, feedback, measures, references) from a credible source. Select N/A if the app component is irrelevant.

Q13 Accuracy of app description (in app store): Does app contain what is described?

- ◯ Misleading. App does not contain the described components/functions. Or has no description (1)
- ◯ Inaccurate. App contains very few of the described components/functions (2)
- ◯ OK. App contains some of the described components/functions (3)
- ◯ Accurate. App contains most of the described components/functions (4)
- ◯ Highly accurate description of the app components/functions (5)

Q14
Goals: Does app have specific, measurable and achievable goals (specified in app store description or within the app itself)?

- ◯ N/A Description does not list goals, or app goals are irrelevant to research goal (e.g. using a game for educational purposes) (1)
- ◯ App has no chance of achieving its stated goals (2)
- ◯ Description lists some goals, but app has very little chance of achieving them (3)
- ◯ OK. App has clear goals, which may be achievable. (4)
- ◯ App has clearly specified goals, which are measurable and achievable (5)
- ◯ App has specific and measurable goals, which are highly likely to be achieved (6)

Q15
Quality of information: Is app content correct, well written, and relevant to the goal/topic of the app?

- N/A There is no information within the app (1)
- Irrelevant/inappropriate/incoherent/incorrect (2)
- Poor. Barely relevant/appropriate/coherent/may be incorrect (3)
- Moderately relevant/appropriate/coherent/and appears correct (4)
- Relevant/appropriate/coherent/correct (5)
- Highly relevant, appropriate, coherent, and correct (6)

Q16
Quantity of information: Is the extent coverage within the scope of the app; and comprehensive but concise?

- N/A There is no information within the app (1)
- Minimal or overwhelming (2)
- Insufficient or possibly overwhelming (3)
- OK but not comprehensive or concise (4)
- Offers a broad range of information, has some gaps or unnecessary detail; or has no links to more information and resources (5)
- Comprehensive and concise; contains links to more information and resources (6)

Q17
Visual information: Is visual explanation of concepts – through charts/graphs/images/videos, etc. – clear, logical, correct?

- N/A There is no visual information within the app (e.g. it only contains audio, or text) (1)
- Completely unclear/confusing/wrong or necessary but missing (2)
- Mostly unclear/confusing/wrong (3)
- OK but often unclear/confusing/wrong (4)
- Mostly clear/logical/correct with negligible issues (5)
- Perfectly clear/logical/correct (6)

Q18
Credibility: Does the app come from a legitimate source (specified in app store description or within the app itself)?

- ☐ Source not identified (1)
- ☐ Source identified but legitimacy/trustworthiness of source is questionable (e.g. commercial business with vested interest) (2)
- ☐ Appears to come from a legitimate source, but it cannot be verified (e.g. has no webpage) (3)
- ☐ Developed by small NGO/institution/specialized commercial business, funding body (foundation money) (4)
- ☐ Developed by government, (5)
- ☐ Developed by a public or private university/academic setting (6)
- ☐ Developed using nationally competitive government or research funding (e.g. CDC, NIH) (7)
- ☐ Developed by hospital/health care affiliate (8)
- ☐ Developed by advocacy group (9)

Q19
Evidence base: Has the app been trialled/tested; must be verified by evidence (in published scientific literature)?

- ○ N/A The app has not been trialled/tested (1)
- ○ The app states that it is evidence based or based on research (2)
- ○ The evidence suggests the app does not work (3)
- ○ App has been trialled (e.g., acceptability, usability, satisfaction ratings) and has partially positive outcomes in studies that are not randomised controlled trials (RCTs), or there is little or no contradictory evidence (4)
- ○ App has been trialled (e.g., acceptability, usability, satisfaction ratings) and has positive outcomes in studies that are not RCTs, and there is no contradictory evidence (5)
- ○ App has been trialled and outcome tested in 1-2 RCTs indicating positive results (6)
- ○ App has been trialled and outcome tested in > 3 high quality RCTs indicating positive results (7)

End of Block: App Quality Ratings

Start of Block: App Subjective Quality

Section E

Q20 Would you recommend this app to people who might benefit from it?

- Definitely yes (I would recommend this app to everyone) (1)
- Probably yes (There are many people I would recommend this app to) (2)
- Might or might not (There are several people whom I would recommend it to) (3)
- Probably not (There are very few people I would recommend this app to) (4)
- Definitely not (I would not recommend this app to anyone) (5)

Q21
Assuming you had recently been assaulted: How many times do you think you would use this app in the next 12 months if it was relevant to you?

- None (1)
- 1-2 times (2)
- 3-10 times (3)
- 10-50 times (4)
- > 50 times (5)

Q23 What is your overall rating of the app?

- One of the worst apps I've used (1)
- Poor (2)
- Average (3)
- Good (4)
- One of the best apps I've Used (5)

End of Block: App Subjective Quality

Start of Block: App-Specific

These added items can be adjusted and used to assess the perceived impact of the app on the user's knowledge, attitudes, intentions to change as well as the likelihood of actual change in the target health behavior.

Section F

Q1
Awareness: This app is likely to increase awareness of the importance of addressing sexual assault and intimate partner violence.

	Strongly agree (1)	Somewhat agree (2)	Neither agree nor disagree (3)	Somewhat disagree (4)	Strongly disagree (5)
Awareness (1)	○	○	○	○	○

Q2
Knowledge: This app is likely to increase knowledge/understanding of sexual assault and intimate partner violence

	Strongly agree (1)	Somewhat agree (2)	Neither agree nor disagree (3)	Somewhat disagree (4)	Strongly disagree (5)
Knowledge (1)	○	○	○	○	○

Q3
Attitudes: This app is likely to change attitudes toward improving sexual assault and intimate partner violence

	Strongly agree (1)	Somewhat agree (2)	Neither agree nor disagree (3)	Somewhat disagree (4)	Strongly disagree (5)
Attitudes (1)	○	○	○	○	○

Q4
Intention to change: This app is likely to increase intentions/motivation to address sexual assault and intimate partner violence

	Strongly agree (1)	Somewhat agree (2)	Neither agree nor disagree (3)	Somewhat disagree (4)	Strongly disagree (5)
Intention to change (1)	o	o	o	o	o

Q5
Help seeking: Use of this app is likely to encourage further help seeking for sexual assault and intimate partner violence (if it’s required)

	Strongly agree (1)	Somewhat agree (2)	Neither agree nor disagree (3)	Somewhat disagree (4)	Strongly disagree (5)
Help Seeking (1)	o	o	o	o	o

Q6 Behaviour change: Use of this app is likely increase/decrease sexual assault and intimate partner violence (or their sequelae)

	Strongly agree (1)	Somewhat agree (2)	Neither agree nor disagree (3)	Somewhat disagree (4)	Strongly disagree (5)
Behaviour change (1)	o	o	o	o	o

Q56 Closing thoughts, comments, summaries:

____

End of Block: App-Specific
